# Mining Personal Data Using Smartphones and Wearable Devices: A Survey

**DOI:** 10.3390/s150204430

**Published:** 2015-02-13

**Authors:** Muhammad Habib ur Rehman, Chee Sun Liew, Teh Ying Wah, Junaid Shuja, Babak Daghighi

**Affiliations:** Faculty of Computer Science and Information Technology, University of Malaya, Kuala Lumpur 50603, Malaysia; E-Mails: csliew@um.edu.my (C.S.L.); tehyw@um.edu.my (T.Y.W.); junaidshuja@siswa.um.edu.my (J.S.); babak@um.edu.my (B.D.)

**Keywords:** data mining, mobile computing, personal data, wearable computing

## Abstract

The staggering growth in smartphone and wearable device use has led to a massive scale generation of personal (user-specific) data. To explore, analyze, and extract useful information and knowledge from the deluge of personal data, one has to leverage these devices as the data-mining platforms in ubiquitous, pervasive, and big data environments. This study presents the personal ecosystem where all computational resources, communication facilities, storage and knowledge management systems are available in user proximity. An extensive review on recent literature has been conducted and a detailed taxonomy is presented. The performance evaluation metrics and their empirical evidences are sorted out in this paper. Finally, we have highlighted some future research directions and potentially emerging application areas for personal data mining using smartphones and wearable devices.

## 1. Introduction

Every day, billions of user-specific data points are generated by personal sensing devices (PSDs), such as smartphones and wearable devices, also known as resource-constrained environments (RCEs) [1]. The *MIT Technology Review* reports 99.5% of newly created digital data remains unanalyzed [2]. This technological advancement presents an opportunity to quantify each second of humane life, allowing information to be obtained by analyzing data from our bodies and daily activities. These personal data can be exploited by data mining algorithms to discover hidden knowledge patterns, which may include frequent activities, classification of physiological data, and clusters of mobile trajectories. Thus, personal data mining techniques are surveyed in this study to set a direction for data analysis in RCEs.

Personal data mining (PerDM) (a.k.a. Personal Analytics and Quantified-Self) is a relatively new concept that is based on data mining techniques used for mining the personal data of users to fulfill their personal needs [3]. The idea deriving the emergence of PerDM is twofold: maximizing the benefits gained from personal data to create well-managed healthy lifestyles while preserving privacy and security [4]. On the one hand, a rapid growth can be observed in the development of data mining technologies and algorithms, as evidenced by quantified-self movement by Kevin Kelly and personal analytics by Stephen Wolfram [5]. On the other, the publicness of the users' personal data in ubiquitous and pervasive environments has raised strong concerns over privacy-preservation and security [4]. At present, computational power and memory volume continues to be expanding, suggesting that PerDM would become more feasible in the near future and attract more research attention [6].

Computational, communicational, and storage resources that are available in a user's vicinity form a new ecosystem called a personal ecosystem (PE). In connection with PEs, not only are PSDs being made resourceful but also providing numerous APIs to maximize the benefits from on-board sensors and device-resident data [1]. Despite the immense growth in micro- and nanotechnologies, PSDs are still restricted in terms of computational power, energy consumptions, on-board memory, and small screen real-estate [7]. Moreover, limited storage capacities and bandwidth are major constraints of PSDs. Nonetheless; PSDs present advantages in terms of mobility, in-hand real-time data processing, and continuous monitoring of user activities. Despite these constraints and limitations, PSDs are being considered as a strong candidate for future data processing systems. Thus, it could be perceived that PSDs can play significant role in PEs to uncover hidden knowledge patterns from daily lifestyle activities and user-specific information [8]. A primary motivation of this study is finding opportunities to maximize benefits from PEs. A number of terms are used in this paper (introduced or imported) and are summarized in Table 1. A more detailed definition is given when each concept is first used in the text.

The main contribution of this paper is presenting a detailed taxonomy of PerDM in RCEs. A comprehensive literature review was performed to elucidate data mining systems from different perspectives. In this study, we presented the details of data sources, design considerations, different application models, and data mining algorithms for PerDM in RCEs. In addition, we illustrated PEs, as well as the constituent components within, and emphasized the resource-scarcity in PEs. Moreover, we explored the latest relevant literature to find the empirical evidences for evaluation criteria used for data mining algorithms in RCEs. To the best of our knowledge this study is the first to address both the hardware and software aspects of PerDM in RCEs.

The rest of this paper is organized as follows: Section 2 provides the background of PEs and related resource-constraints. Section 3 presents a taxonomical discussion of relevant concepts of PerDM in RCEs. The evaluation criteria and their evidence in the literature are also presented in this section. Section 4 discusses the role of PerDM in PSDs to balance among personalization, privacy and security in RCEs. Section 5 is about application areas and open research issues. Finally, the article is concluded in Section 6.

## 2. Background

### 2.1. Personal Ecosystem

**Figure 1.**
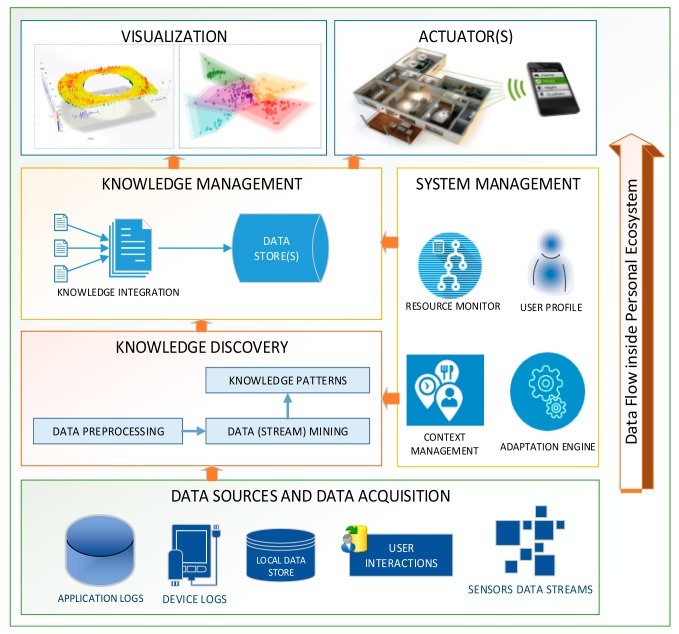
Data lifecycle in PEs.

A notable advantage of PSDs as data-processing platforms is their ability to reduce the computational burden at remote facilities by performing initial data processing at the users' locality. As depicted in Figure 1, general data processing and knowledge management in PSDs are based on six main modules: (a) data sources and data acquisition; (b) knowledge discovery process; (c) knowledge management; (d) system management; (e) visualization; and (f) actuators.

#### 2.1.1. Data Sources and Data Acquisition

PSDs enable the handling of heterogeneous data streams from both sensory and non-sensory data sources. On-board sensing data sources include a huge variety of sensors for sensing contextual and physiological information, locations, and environments [1]. Details of these data sources are listed in Table 2. User interaction with PSDs and device resident log files generate non-sensory data streams, which create multifaceted data streams. Numerous APIs and data collection tools are available for acquiring these data streams. For example, Android SDK [9] provides a sensor manager that can obtain data from on-board accelerometers, compasses, GPS, magnetometers, and cameras. Similarly, the mobile sensor data-processing engine (MOSDEN) is a highly sophisticated data collector developed for opportunistic sensing in Internet-of-Things (IoT)-enabled environments [10]. MOSDEN acquires application-independent data and provides cross-platform data sharing, thereby resulting in a more dynamic and useful data acquisition. In addition, data preparation is performed at this layer by applying windowing models or distributing the data in equally sized chunks for further online data analysis.

#### 2.1.2. Knowledge Discovery Process (KDP)

In PSDs, knowledge discovery involves three steps: (1) data preprocessing is performed; (2) data (stream) mining algorithms are applied to uncover hidden patterns; and (3) interesting knowledge patterns are sorted after pattern evaluation [11]. At the first step, preprocessing tasks include data fusion and pipelining strategies, outlier detection, anomaly detection, dimensionality reduction, and feature extraction techniques. At the second step, data mining or data stream mining algorithms for classification, clustering, and frequent pattern mining are applied on the preprocessed data to extract knowledge patterns. The choice between data mining and data stream mining solely depends on the application requirements and the need for online (data stream) or offline (data mining) data analysis. Lastly, the discovered patterns are evaluated against different measures of interestingness, and on the basis of which, the decision whether to store or discard is made at this stage. For example, the interestingness measure for classification could be either the minimum number of classes or the perceived level of accuracy. Similarly, for cluster evaluation, the number of clusters, their centroids, and the distance among them can be used for pattern evaluation. The pattern evaluation techniques are further discussed in detail in Section 3.3.

#### 2.1.3. Knowledge Management

Knowledge patterns are aggregated, summarized, or integrated for further utilization. Then, these summary datasets are forwarded to the local storage. Onboard storage elements and removable SD-cards are typically used for local storage in PSDs. In case of unavailability of sufficient local storage, the datasets are sent to external environments for permanent storage. These external environments include remote data processing systems in clouds, grids, or on the Internet.

#### 2.1.4. System Management

PSD-based data processing systems should be adaptive; that is, they should consider contextual information and resource monitoring [12]. The adaptation engine plays a critical role in executing seamless knowledge discovery processes inside PSDs without compromising the overall performance of mobile devices. In addition, a key function of the adaptation engine is enabling PSDs to process maximum data locally. Therefore, the adaptation engine collaborates with the context manager and on-board resource monitor modules. The context manager provides information about location, activities, and device usage status, among many others. Meanwhile, the resource monitor provides information about on-board available computational (CPU, RAM, ROM), communicational (Wi-Fi, Bluetooth, BLE, Wi-Fi direct), and storage (local storage, SD-card) resources. The adaptation engine periodically executes pre-defined rules for adaptation on the basis of dynamically changing contextual and resource-related information.

Systems manage user profiles to address privacy- and personalization-related challenges. The vulnerability of mobile devices in ubiquitous and pervasive environments has brought about many serious privacy concerns [13]. User profiling or modeling in PE helps to mitigate these privacy risks by enhancing user control over their personal data and patterns. An alternative option for privacy preservation is the utilization of privacy-preserving data mining algorithms that anonymize personal data before being shared in external environments. An added advantage of user profiling is that mobile users are provided with personalized services. In general, service providers implement mass customization to meet the needs of large groups of users; but by enabling privacy-preserving profile sharing, effective personalized services can be tailored by these providers.

#### 2.1.5. Visualization

Knowledge visualization in PSDs can be either local or remote. Local visualization at small screen real-estate in PSDs is useful for basic visualization. This limitation sometimes necessitates remote visualization using more resourceful PSDs or external environments, because detailed and graphic intensive visualization are not usually provided in most PSDs [7]. Currently graphic-intensive PSDs are emerging but they are not very useful for real-time data mining systems displaying continuous patterns and utilizing immense battery and computational powers.

#### 2.1.6. Actuators

Software modules serve as actuators for facilitating interactions with external systems. These external systems can be IoT-enabled smart homes and other similar smart spaces or remote data processing systems based on clouds, grids, and other Internet-enabled large infrastructures [5].

### 2.2. Constraints in Personal Ecosystem

Given the small size, portability, and mobility requirements of users, PSDs are limited in terms of battery power, computation, communication, and visualization facilities. Major constraints of PSDs include bandwidth, CPU cycles, memory, storage, visualization, mobility, and connectivity [14,15]. Although advancements in micro- and nano-chipset technologies have empowered PSDs, these resource-constraints need to be considered in the development of data mining algorithms. A previous study has argued that resource and context awareness enable algorithms to autonomously adapt in volatile execution environments without any external interference and commands [16].

First, a major requirement of data mining algorithms is efficient energy utilization (EEU) because of the limited available energy resource in PSDs. EEU in smartphones is challenged by on-board sensing, feature-rich datasets, and continuous computational requirements with support for high-resolution graphics and multimedia services [17]. A study has shown that EEU can be improved by understanding the users' interaction with mobile phones and by switching off irrelevant sensors and communication channels [18,19]. Another study has presented detailed information on human–battery interaction in a smartphone's context, in which major behavioral, usage, and technical implications on EEU were highlighted [19]. Therefore, these aforementioned findings should be considered in devising a design strategy for data mining applications in PSDs.

The decision of what, when, and how much data to analyze varies by nature and requirements of the data mining algorithms. Therefore, a designer should address this issue on a case-by-case basis depending on the application requirements. For example, for real-time analysis, a user must either immediately perform local analysis or offload to some external computational infrastructure. Similarly, some applications may require periodic analysis and create a space for offline analysis during nighttime or when the PSD is charging. Another form of EEU is adapting algorithms with available resources in PSD. Such adaptation can be in terms of computational cycles, the sampling rate of sensor data, or the size of sliding windows [20–22]. The adaptation strategy is generally devised by establishing a trade-off between EEU requirements and the accuracy of knowledge discovery algorithms.

EEU is a more critical issue in collaborative PSD environments, where the allocation strategy for data mining tasks can affect the overall performance. Researchers on EEU have used energy-based scheduling rather than conventional time-based scheduling schemes [14,23]. The proposed design utilizes localized clusters of mobile devices to execute data mining tasks using energy-aware task scheduling. The heuristics-based scheduling algorithm works in two task assignment phases: (a) it schedules the task in the local cluster of request origin; and (b) otherwise, it assigns the task to the most suitable node in the overall network by considering the energy consumption of both the communication and computation of tasks.

Second, another major requirement of data mining in PSDs is bandwidth utilization cost (BUC) because of the financial and energy utilization burdens in PSDs. BUC can be lowered by adopting efficient computation offloading, local data processing, adaptation, and compression–decompression techniques. Various computation offloading schemes are available for performing remote data processing in PSDs. For example, a previous study has investigated whether to perform static or dynamic offloading by conducting a detailed survey on the implications of computational offloading on performance and energy consumption [24]. In addition to this, different kinds of PSDs, mobile applications, and computation offloading infrastructures are discussed in detail in this study.

BUC can be lowered by performing local data analysis; however, the overhead cost can affect the EEU in PSDs. Alternately, raw data can be compressed in PSDs after a trade-off is established between EEU cost locally and BUC for computation offloading. After a thorough analysis of application partitioning schemes and mobile cloud computing application models [24,25], we can formulate a computation offloading strategy as shown in Algorithm 1.

**Algorithm 1** Computation Offloading Strategy
1:**if** (cost (EEU) > BUC)2:{3:Data ← Compress (raw-data);4:Send (Data, remote_system);5:}6:**else** Run7:(local_data_mining_algorithm, raw-data).


The offloading strategy works when the EEU cost in the mobile device exceeds the BUC. In this case, raw data is first compressed and then offloaded to cloud services for further processing. Alternately, local data mining algorithms are applied over raw data for local knowledge discovery.

Third, CPU cycles, which represent the computational power, are limited in PSDs. Therefore, data mining algorithms should be designed accordingly. The design considerations for such algorithms include light-weight algorithms, adaptive algorithms, and scalable algorithms. Light-weight algorithms are developed by considering key analytical requirements, and results are approximated accordingly [26]. Adaptive algorithms are designed such that they are reconfigurable and adjustable according to the available computational resources in PSDs [12]. Algorithms are considered scalable when a number of computational tasks are performed inside PSDs while other tasks are outsourced to other PSDs in the locality [20].

Fourth, memory is another key constraint in PSDs, particularly in smartphones where memory is shared between multiple applications and processes that provide support to online and offline data analysis. Energy-efficient techniques for tuning size, attributes, and results-related parameters of data are generally helpful in effective memory management in PSDs; conversely, learning-intensive algorithms pose serious challenges for memory efficiency [23]. An alternative approach is to perform offline or incremental learning to effectively utilize on-board memory resources in PSDs [21,22,27]. The learning methods are explained in detail later in Section 3.1.5. In addition, garbage collection should be considered an intrinsic feature at every phase of data mining in PSDs to attain high availability of memory resources. Additional memory optimization techniques are also helpful. For example, memory resources can be optimized by closing inactive applications, deleting application and device log files, as well as pausing irrelevant background processes.

Fifth, storage is relatively scarce resources in PSDs. Researchers have studied the effect of storage on application performance in mobile phones [28]. The authors argue that internal storage (flash memory) showed 187% variability in performance compared with 2040% variability in applications running on external storage (SD-card and USB devices). Storage is considered a key constraint in PSDs because of its huge impact on application performance, specifically in four ways: (a) runtime; (b) launch time; (c) concurrent applications; and (d) CPU utilization. Moreover, performance is also affected by read/write operations in sequential and random manners. Therefore, data mining algorithms developed for PSDs should consider these drawbacks to maximize optimization and performance.

Sixth, visualization poses a major drawback for PSD-based data mining systems because of the small, or sometimes even absent, screen real-estate. In the case of wearable devices and body sensor networks (BSNs), knowledge patterns are usually transported to a central processing system with visualization facility or to an integration device in the vicinity of the sensing devices. Smartphones generally have sufficient screen size for basic visualization facilities; however, detailed graphics-rich visualization requires the use of larger multimedia display screens, such as that of tablets, desktops, or overhead projectors. An adaptive visualization approach has been proposed to effectively deal with on-screen clutter of results from data stream mining algorithms [7]. The research on knowledge visualization in PSDs is still at its early stages, and many issues related to adaptation, fast results handling, and scalability with volatile data rates remain to be addressed.

Lastly, PSDs are always moving and switching between different networks, thus indicating the need for efficient mobility management policies for PSD-based data mining systems. Data mining systems must be capable of tracking the devices for efficient data transfer between data sources and data mining systems. In some cases, the distant nature of mobile devices causes latency, noise, and incomplete data transmission. A four-dimensional design framework has been suggested to discuss the application mobility challenges associated with temporal, spatial, entity axes, and an extra axis for design concerns [29]. Moreover, connectivity is also a challenge in PSDs because of the weak Wi-Fi signals receptions as compared to laptops and other powerful portable devices. In addition, conventional Bluetooth connections consume large energy, emphasizing the need for Bluetooth Low Energy (BLE) devices. Researchers have presented a performance study on the effect of Wi-Fi and Bluetooth on the power and throughput delay of smartphones [30]. They posit that Wi-Fi is the better choice for design considerations because of its better energy utilization and throughput performance.

## 3. PerDM in Resource-Constrained Environments

### 3.1. Taxonomy

**Figure 2.**
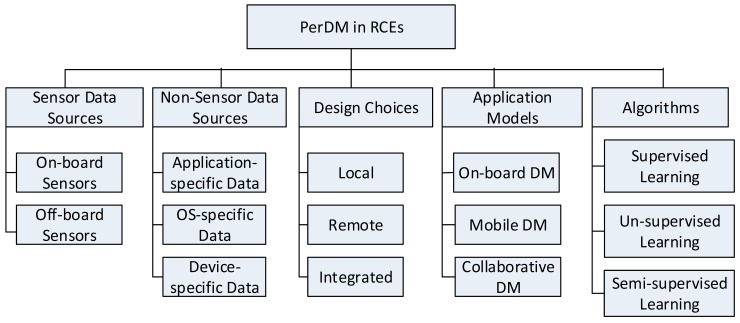
Taxonomy of PerDM in RCEs.

In this section, we present taxonomy of PerDM in RCEs in terms of data generation, design choices, application models, and algorithms (Figure 2). Furthermore, we describe the empirical evidence for data mining algorithms strictly applied in RCEs for PerDM and highlight some application areas and related challenges.

#### 3.1.1. Sensor Data Sources

Sensor configurations in PSDs vary in terms of: (a) locality; (b) placement; and (c) modality. The locality of sensors [31] in PSDs is further referenced in two forms: (a) sensors configured on the same motherboard as computational components, such as CPU, memory, and storage facilities; and (b) sensors placed in remote wearable devices. Although useful for real-time data processing systems, local sensors-based PSDs are disadvantaged by heat dissipation and energy consumption. Nonetheless, data collected from off-board sensors-based PSDs are prone to noise and communication protocol hurdles. Thus, deciding where to place sensor on body locations presents additional challenges for PSD-based data mining systems.

In PEs, sensors can be configured in multiple ways [31]: (a) on-body through wearable devices; (b) off-body through smartphones and other smart spaces; or (c) sensors implanted inside the body.

Selecting the best location for the sensors in PEs is critical in obtaining exact readings and noise-free data collection. For example, a study shows that PSDs placed in the front and back of a pants pockets produce different results [21]. Therefore, sensor placement must be considered in any PSD-based data mining system.

Modality constraints in PSDs [32] can be categorized in two forms: (a) to sense inside PEs only; or (b) to gather data from external environments as well. The fusion of data points from both modalities enable contextual information from external environments to be maintained. Consequently, different sensor configurations result in heterogeneity and complexity in data enabling feature-rich PerDM systems. A number of commonly known data sources in PSDs are presented in Table 2. Notably, wearable devices generate sensor data streams, whereas smartphones may handle non-sensor data streams as well.

#### 3.1.2. Non-Sensor Data Sources

In addition to sensor data streams, a huge variety of data streams is present in smartphones. These non-sensor data streams can be categorized as: (a) device-resident; (b) application-resident; or (c) user-interaction based data.

As a powerful PSD, smartphones store a number of log files to maintain: (i) communication and (ii) device-status related periodic information. Communication-related information includes Wi-Fi and Bluetooth scans as well as data about cellular networks and nearest towers. Status logs store information related to battery, operating system, and device hardware. Thus, a huge amount of data points can be gathered from device-resident information.

Mobile applications gather a variety of user-related information. For example, mobile web browsers maintain cookies to store user credentials and personal login information for social networks and mail-service providers. The sensitivity of this information suggests the need for privacy-preserving data mining systems.

Moreover, a user's interaction with smartphones, including the use of on-screen keyboards, microphone, and video cameras, is the key driver of non-sensor data production in PSDs. Non-sensor data sources and their data types is presented in Table 2. Several data mining systems and algorithms for PSDs have been proposed in the literature to address evolving data source heterogeneity in PE. We will discuss these algorithms in detail later in this section.

#### 3.1.3. Design Choices for PSDs-Based Data Mining Systems

Trade-offs between on-board computational resources and the cost of computation offloading to remote data processing systems should first be analyzed before personal data mining systems are designed. PSD-based data mining systems are generally designed in three modes: (a) local; (b) remote; and (c) integrated. Some of these systems either analyze data locally or offload them for remote processing, whereas some systems work in both modes.

**Figure 3.**
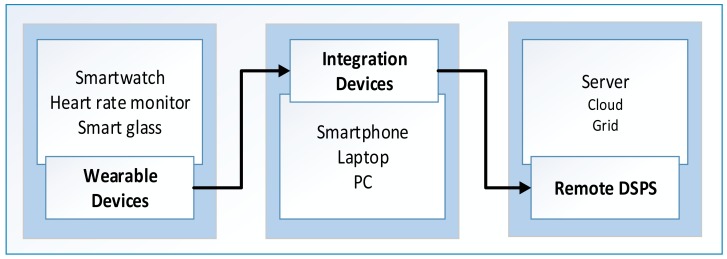
Data flow from PSDs to remote DSPS.

a) Local Processing
  The selection of local processing mode depends on the availability of on-board computational and battery power near the sensors [33]. Local processing is very effective for in-situ analysis over continuous fast sensor data streams. These systems are further configured as field programmable gate arrays (FPGA)-based and CPU-based infrastructures.
  FPGA-based systems are reprogrammable, reconfigurable, and customized for specific purposes [34]. These properties make FPGA-based systems ideal for real-time processing systems. Comparatively, although the general-purpose nature of CPU-based systems makes them slower as compared to FPGA-based systems, the former can provide variety of operating systems and programming APIs for application development. Deployment of general-purpose data mining algorithms, with the use of these resources, demonstrates that CPU-based systems are a better candidate for modern PSDs.
b) Remote Processing
  The need for remote processing mode arises when data sources and processing systems are located apart from one another and local processing systems are either unavailable or have insufficient resources to meet computational requirements [35]. Remote data stream processing systems (DSPS) include PSDs and other large computational infrastructures, such as clusters, clouds, and grids. Most PSDs rely on remote DSPS for computation offloading and knowledge discovery because of the limited on-board resources. A hierarchy of PSDs is being used in modern DSPS systems, as depicted in Figure 3, to maximize the computational benefits from the nearest PSDs before computation offloading to large DSPS. For example, mobile cloud-lets are used for provision of computational facilities to PSDs in the locality [36]. Similarly, smartphones are being used as integration devices (IDs) for modern wearable sensing devices.
  The discussion on remote large-scale DSPS requires the examination of widely accepted tools and technologies. These DSPS are used for various heterogeneous data stream processing and enable large-scale *ad-hoc* queries over high-speed data. In addition, remote DSPS support multiple programming languages, including Java, C++, and Python. There is a long list of emerging analytical tools but we limited our discussion to the stable versions of these DSPS. A summary of some of these systems is presented in Table 3.
c) Integrated Processing
  Integrated processing mode is the combination of both local and remote data processing systems [20,27,50]. In this configuration, initial data processing is performed in near-sensor PSDs, and results are transported to remote DSPS for further analysis. This approach not only balances the computational burden between PSDs and remote DSPS but also helps to reduce energy consumption during data transmission and financial costs for computation offloading to remote DSPS.
  The configuration of integrated processing mode is made in different modes: (a) between PSDs only; and (b) between PSDs and remote DSPS. Therefore, this configuration enhances the versatility and design choices for system designers. For example, PDM is purely based on PSDs and their combinations, whereas other systems transport data from smartphones to mobile clouds [50]. Other examples [51] of PSD-based configurations are Samsung's Galaxy S5 and Gear2 as integrated processing systems where initial analysis are performed at wearable devices while the rest of the tasks are executed at the host device end.

#### 3.1.4. Application Models for PSDs Based Data Mining Systems

The discussion on PSD-based PerDM would be incomplete without providing an overview of some widely-accepted practical software and frameworks. The scope of this section is limited to some renowned and innovative configurations of stream execution models in PEs. Additionally, we provided examples of practical software for each of these execution models and articulated some core features of these software and frameworks. The rest of this section discusses PerDM in on-board applications, mobile devices, and collaborative environments.

a) On- (device) board Data Mining
  The increasing variety in sensors and developments in mobile computing platforms is the key factor in the adoption of on-board data stream mining [33]. Numerous on-board data mining systems have been successfully implemented to sense earth, climate, weather, health, spacecraft, robotics, and other physical and virtual sensing environments. However, at present, on -board data mining systems are facing the problem of low computational resources, that is, they have to handle noisy data as well as incompleteness and inaccuracies during data measurements. Moreover, geo-referencing (*i.e.*, a priori information about location of sensors and sensing platforms) has become an essential requirement in on-board data mining applications.
  On-board data stream mining algorithms are being designed for both FPGA-based and CPU-based systems, although the latter comprise the better choice in most of the scenarios. FPGAs are reconfigurable and provide more computational resources and bandwidth because of their energy-efficient behavior [34]. The downside of FPGAs is that a new hardware must be designed or re-programmed for each algorithm. Examples of FPGA-based data mining techniques are their recent applications in micro- and nano-robotics [52]. Alternatively, CPU-based systems are general processing architectures; thus, they consume more energy and perform slower because of recursive scheduling constraints in the system. However, deployment and configuration of data mining algorithms in CPU-based systems is not a hectic and redundant task as compared with FPGA-based systems.
  CPU-based on-board data mining systems, strictly applied in RCEs, are often discussed in data mining literature. Open Mobile Miner (OMM) [12], VEDAS [53], and MineFleet [54] are some examples of on-board mobile DSPS. The scope of CPU-based data mining systems lies in both on-board and mobile data mining. Hence, the details of these systems are presented in the next subsection.
b) Mobile Data Mining
  The applications models involving mobile phones as data-generating elements are based on three schemes: (i) mobile interface; (ii) on-board CPU; and (iii) client-server.
  In the first approach, mobile interface, mobile applications only provide interfaces and the data mining tasks are performed on the back-end computational infrastructures [20]. In the second approach, the on-board CPU-based mobile data mining, data mining tasks are performed locally on the mobile devices. In the third approach, the client-server approach, some data mining tasks are performed in mobile devices, while some tasks are performed at back-end server [55].
  Each of these approaches has its own strengths and limitations. For example, there is greater communication overhead in mobile interfaces than in on-board CPU-based data mining systems [20]. Moreover, data visualization and low latency are the positive aspects of on-board CPU-based data mining system, but these may be lacking in mobile interfaces. On the one hand, processing, memory, battery power, and storage are the key considerations in on-board CPU-based data mining systems; on the other hand, these are not major issues in mobile interfaces.
  Considering the popularity of mobile phones and smartphones, some application models and frameworks have been proposed for mining data streams generated and/or sensed by these devices. For example, a component-based framework, Mobile Smart Archive (SMA), has been proposed for collecting and mining sensor data streams on mobile devices [56]. SMA was developed using C++; thus, the project was not widely accepted due to Java-based implementations that were not yet possible due to the lack of JVM-supported mobile devices at that time. Hence, SMA was not sustained for a long time.
  Another example is the development of a web service-based data mining system to mine databases by accessing data through mobile phones and displaying results back on these mobile phones [55]. In this system, the data are acquired by web servers from different data sources and then stored on mining servers. A MIDlet, deployed on a mobile phone, sends requests for particular data mining tasks, after which the mining server invokes the relevant web service and returns the results back to the mobile phone after completion. Although it was a good approach for that time, there were issues related to visualization and communication burden, because of limited bandwidth on the mobile phones, which demanded an effective data stream mining solution.
  A recent development is OMM, a generic tool for mobile data mining that senses the environment and performs local data mining tasks [12]. Key components of OMM architecture are data sources, data stream capture, adaptation engine, library of data stream mining algorithms, resource monitor, and visualization library. In addition, the researchers [57] proposed Context-Aware Real-Time Open Mobile Miner (CAROMM) for mobile crowd-sensing. The framework reduces the energy consumption and bandwidth utilization during data communication over mobile networks. The CAROMM operates using data mining algorithms in mobile devices and performing initial data processing at user end to reduce the computational burden over clouds. Similarly, Mobile WEKA is another general data mining tool developed for android devices; it supports classification, clustering, and association rule mining algorithms [58].
c) Collaborative Data Mining
  Requirements on energy efficiency and limitations in computational power have resulted in the collaborative performance of data mining in *ad-hoc* mobile networks. There are two design considerations for these *ad-hoc* mobile networks meant for collaborative data mining. First, the devices in same locality are connected in peer-to-peer configurations. Second, an *ad-hoc* mobile cloud is established at run time to fulfill the processing needs of subscribed mobile users. Pocket Data Mining (PDM) is an example of the first configuration, yet no study has examined the second configuration in the literature [20]. Thus, we limited the current study to PDM, which is presented in the next paragraph.
  PDM is a collaborative data stream mining framework based on mobile devices, agent technology, and data stream mining algorithms. The basic motivation behind PDM is the opportunity to utilize mobile devices collaboratively for knowledge discovery in ubiquitous environments and to address the resource-constraints and energy efficiency challenges posed by conventional mobile data mining systems. PDM is suitable for a range of application domains including health, safety, traffic management, policing, crowd control, crises and riot managements; however, this approach comes with security- and privacy-related concerns that could be very serious in some situations. A complete monograph about PDM discusses the motivation, framework, experimental setups, results, and research challenges associated with collaborative data mining [20].

#### 3.1.5. Data Mining Algorithms

The core of taxonomy is the exploitation of data mining algorithms in RCEs. The detailed literature review of these algorithms is presented in Section 3.2 of this paper. Here, key learning methods, such as supervised learning and unsupervised learning are defined and presented. In addition, we have highlighted the importance of semi-supervised learning, which could be a preferable choice for PerDM in PSDs, in uncovering knowledge patterns when moving from known spaces to unknown spaces.

Machine learning algorithms, also called learning models (LM), play a significant role in PerDM. Although the selection and deployment of these models is difficult owing to on-board available resources, LMs are widely adopted in PSDs as well. Learning schemes for PSDs vary in two dimensions (*i.e.*, off-line and on-line), and learning process takes place linearly or incrementally. In case of off-line learning, the learning models are first trained out-of-PSD and then used inside PSDs, thus compromising accuracy as well as personalization at user-end [21,22]. Alternately, on-line learning provides more accurate and personalized models, but less EEU because of computation intensities inside PSDs [59]. Meanwhile, linear learning is more computation-intensive compared with incremental or ensemble learning. Hence, the choice of learning algorithm significantly affects the overall performance of PSDs. A precise discussion of three widely used learning modes is being presented by considering resource constraints and computational requirements of learning schemes in PSDs.

The design consideration for learning algorithms include support to heterogeneous and redundant data, input space dimensionality, trade-off between “bias” and “variance” in input space, noise in output spaces, linearity and non-linearity of feature vector space, functional complexity, and amount of training data [60].

**Figure 4.**
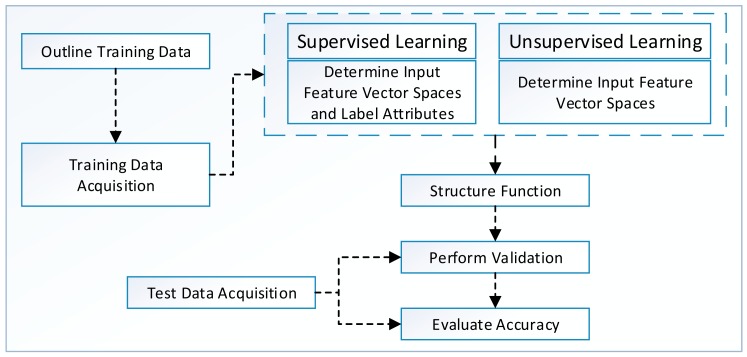
SL and UL algorithm development process.

**Figure 5.**
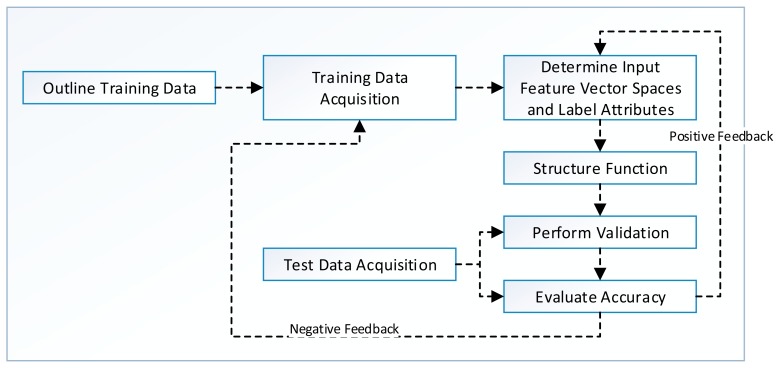
SSL algorithm development process.

a) Supervised Learning (SL)
  One of the most common tasks in data mining is to build models for the prediction of an object's class on the basis of its labeled attributes. A classification or regression model is usually trained for class prediction on large data sets. Model training is done using supervised learning, which allows for manual labeling of data points so that classification algorithms can predict similar unobserved data [61]. An overview of SL algorithm development process is presented in Figure 4. The designer first outlines the type and amount of training data that could help in building prediction models. The training data are acquired from multiple data sources discussed in Sections 3.1.1 and 3.1.2, and feature extraction is performed for dimensionality reduction. In addition, instances are labeled manually (by user) or automatically (by application).
  The SL algorithms work by taking A (a set of input spaces with a_i_ feature vectors and L_i_ labeled attributes) and invoking a learning function that maps A to B (set of output spaces). The selection of function affects the overall performance of the SL algorithm. The evaluation of SL algorithm is performed using cross-validation, hold-out, prequential or leave-one-out techniques. Finally, the model is tested for accuracy using different evaluation criteria. A detailed discussion of these performance evaluation criteria is made later in Section 3.2.
  Formally, SL algorithm works under some assumption or bias for better predictions in unseen test environments. For example, smoothness assumption states that if two points, P_1_ and P_2_, are closer to each other in a training dataset, it is most likely that they will be closer in test data as well. In addition, an algorithm has high variance in SL settings if it predicts different output values when it is trained with different data sets; it is considered biased when it predicts correct results with systematically incorrect input spaces. The prediction error (*P̂_e_*) of a learned classifier is the sum of bias (β) and variance (σ^2^) denoted as *P̂_e_* = β + σ^2^. The trade-off between β and σ^2^ is that LM must be flexible with low β value so that it can fit the data well, but the high flexibility in LM also increases σ^2^ value. Therefore, a good SL algorithm provides a mechanism (automatic or manual) with which to adjust this trade-off between β and σ^2^ and prevent overfitting of the model [61,62].
b) Unsupervised learning (UL)
  The absence of class labels in data leads toward the discovery of new groups using unsupervised learning techniques. Given the large data sets or streams with multiple attributes, the conventional SL algorithms require a great number of computational powers, making it difficult to manually handle all the grouping activities. The algorithm development process of unsupervised learning methods, as depicted in Figure 4, is the same except the labeling of data. In unsupervised learning, the definition of training data and its acquisition is the same, but the input features vector space contains only unlabeled data. The LM is trained and evaluated on the basis of input features vector space and finally tested using separate test data. The primary assumption for unsupervised learning algorithms is that all data points are identically and independently distributed to define a (*n* × *d*) matrix. UL is initially used for density estimation, but now it is equally being adopted for outlier detection, clustering, quantile estimation, and dimensionality reduction [60].
  A few examples of clustering algorithms using unsupervised learning schemes are exemplified by the following applications: Adjustable Fuzzy Clustering (AFC) for activity classification in wearable BSN environments; Light-Weight Clustering (LWC) for energy efficient mobile crowed-sensing; Time-Based Clustering (TBC) for efficient automatic navigational location prediction; k-mean clustering for dimension reduction [63] and energy efficient complex activity recognition; and Gaussian Mixture Model (GMM) for stress classification [57,64–67] using smartphones. The applicability of these schemes proves that clustering using UL is a good candidate for PSD-based data mining systems.
c) Semi-supervised learning (SSL)
  SSL plays an intermediate role between SL and UL. Both the scarcity of labeled data and the extensive labeling efforts are the main bottlenecks of SL algorithms. SSL extends SL by handling unlabeled data as well. Yet, SSL still needs human-intervention but reduces the effort of manually labeling the data. Moreover, SSL is equally exploited in other forms of data mining algorithms, such as clustering and regression. SSL algorithms work best under certain assumptions, and some of these known assumptions include smoothness assumption for classification algorithms, cluster assumption for interrelationship between cluster points, and low density separation assumption for dimensionality reduction algorithms. The assumptions with higher certainty level help develop better predictive models with higher accuracy. Alternately, poorly modeled assumptions can reduce the performance of predictive models [68,69].
  The rest of the algorithm development process depicted in Figure 5 is the same as SL and differs only in feedback propagation in LM. The model is given positive feedback and is updated with labels of accurately predicted input vectors. Meanwhile, negative feedback is sent for re-training to training datasets. SSL algorithms work by first establishing a hypothesis from labeled data and then modifying or prioritizing the hypothesis using unlabeled data. For example, an SSL algorithm takes both A (labeled data) and B (unlabeled data) as input. Using A, it then builds a hypothesized model called LM (A), then it processes B and based on the assumptions, it modifies or ranks LM (A). The new model is called LM (A + B), which means that it can handle both labeled and unlabeled input instances [68].
  SSL could be either transductive or inductive in nature. The transductive learning can only handle the known data points. Alternately, inductive learning enables one to handle unseen data as well. Some commonly used SSL methods include transductive SVM, co-training, self-training, and graph-based methods. An overview of existing literature reveals that there is no SSL method that can be categorized as the best, but the authors [70] recommends a checklist for method selection. They argued that EM with generative models may be a good choice for clustering algorithms. Similarly, co-training is appropriate for two set features, graph-based methods could be used for feature similarities, and self-training methods are best for complicated supervised classifiers. A detailed literature review of these methods can be found in [70] for interested readers.

### 3.2. Literature Review of PerDM Algorithms for PSDs

The comprehensive literature review reveals that classification algorithms are widely accepted for PerDM in RCEs. Supervised learning—best for controlled experiments—is the main basis for the selection of classification algorithms. Moreover, On the one hand, numerous clustering algorithms are being exploited mainly using unsupervised learning methods, but the limitation of insufficient resources is a bottleneck that hampers the maximization of these algorithms. On the other hand, there is a lack of frequent pattern mining techniques in RCEs because of extensive memory requirements for candidate generation and large tree structures. The evidences of the exploitation of these algorithms are presented in the following sections.

a) Classification
  Three types of training models are used for classification in RCEs: (a) universal, a single model that is used for all type of users; (b) personalized, in which a model is trained for each individual; and (c) adaptive, a model that starts with a universal scheme but gradually adapts and becomes personal for each user [64]. Each modeling technique, however, has disadvantages. For example, the universal model is a single model for all users; therefore, the need for more accurate predictions arises because of differences in behavioral and physiological patterns of users. Personalized models are more accurate but require manual labeling for each activity. Finally, adaptive models must be self-trained and manually labeled at both times. Of the three, the universal modeling scheme is used in most of the studies in extant literature.
  A variety of classification and feature extraction techniques from tree-based structures; neural and Bayesian networks; and statistical, probabilistic and regression models; and so on, are exploited in PSDs. For example, tree-based models, such as Hoeffding Tree (HT), Random Forest (RF), Best-First Tree (BFT), J48, and C4.5 have been used for activity recognition, energy efficiency, physiological data analysis, personalization, privacy and adaptation, stress classification, complex activities analysis, and intelligent distributed classification [22,26,64,65,71] in PSDs.
  Similarly, neural network-based models, such as Artificial Neural Networks (ANNs) and Multi-Layer Perception (MLP)-based NNs, are used for fall detection, energy-efficient activity recognition, WSN-based activity recognition, and simple and complex activity recognition [21,65,71,72]. Statistical classifiers, including Support Vector Machines (SVM), Quadratic Discriminant Analysis (QDA), Linear Discriminant Analysis (LDA) and k Nearest Neighbors (kNN), are implemented for activity recognition, injury rehabilitation, physiological data analysis, optimized energy consumption, stress profiling, physical activity recognition, discrimination between stress and cognitive load, real-time activity recognition, and application usage prediction in mobile phones [65,73–79].
  Moreover, several regression models have been used in numerous studies, including Localized Logistic Regression (LLR), Nonlinear Logistic Regression (NLR), and Maximum Entropy (Max Ent) for energy efficiency and intelligent context-based personalized services; Bayesian Network and instance-based algorithms like K-star (K*) for simple and complex activity recognition; and rule-based models like Decision Table (DT) for activity recognition [26,65,71,77,80]. Finally, probabilistic models, such as Naive Bayes (NB), are widely adopted for distance estimation among Wi-Fi users, distributed classification, “callee” recommendation using personal and social contexts, physiological data analysis, personalization, privacy and adaption, and activity recognition [26,59,65,71,73,81–84]. Such a wide-scale adoption of classification algorithms has encouraged researchers to explore new opportunities for PerDM in PSDs.
  The comparative analysis of classification algorithms shows that accuracy is the basic criterion for algorithm selection [62]. The absence of a universal algorithm that works on every dataset imposes the challenge of selecting the best algorithm for a proposed solution. Similarly, the proposed PSD-based data mining system is designed after evaluating some potentially related algorithms. However, there may be a trade-off between accuracy and required computational complexity due to resource-related constraints in PSDs. The study shows that tree-based algorithm (*i.e.*, C4.5) and statistical classifier (*i.e.*, SVM) are amongst the classifiers with the best accuracy in majority of the data sets.
  The accuracy of a classifier depends on the number of instances, attributes, and classes to be predicted [62]. In addition, the application of Principal Component Analysis (PCA) affects performance given that a number of instances and variables negatively affect performance. Meanwhile, PCA variance, the application of PCA, and the number of target classes and nominal variables have positive effects on performance. Moreover, model building time is directly related with number of instances and data sparsity, but accuracy can be improved by efficient handling of noisy data. Thus, the prediction accuracy of classifiers in PSDs is dependent upon data size, number of attributes, preprocessing techniques, and data sparsity.
  The critical analysis of numerous studies relating PerDM in PSDs, as presented in Table 4, shows that the performance of same classification algorithms varies in different environments. For example, in case of tree-based algorithms, C4.5 [71] performed best with more than 90% accuracy compared with NB, but in another study [59], NB performed best with 86% ± 3.9% as compared with 85.9% ± 2.5% of C4.5; furthermore, NB consumes 71 KB less memory than C4.5. Similarly, in some other studies [65,73], SVM outperformed NB in all cases.
  The variety in sensor configurations, device models, on-line and off-line learning, batch and stream analysis, and scheduled and real-time analyses can make a significant difference in the classification results. For instance, differences of latency in local on-board sensors and off-PSD sensors can lead to noisy and unbalanced data, thus affecting the prediction accuracy. Likewise, the resource constraints in real-time system can lead to false and inaccurate predictions [72].
b) Clustering
  Clustering using unsupervised learning schemes creates multiple groups or clusters of highly similar or dissimilar data points. The assessment of such similarities and dissimilarities depends on the attribute values and distance measured from the cluster centroids. Different variants of data clustering techniques include hierarchical, spectral, subspace, and density-, centroid-, and constrained-based techniques. The choice of these techniques solely depends upon the type and nature of data to be clustered as well as the application requirements. However, clustering algorithms are not widely adopted in PSD-based data mining systems due to high and sometimes unlimited computational requirements. In addition PSDs face the challenges of dealing with concept drift and high dimensional noisy data streams in ubiquitous and pervasive environments.
  Clustering algorithms in literature, as summarized in Table 5, ascertain that AFC can be used for the provision of incremental learning in PSDs. For example, researchers proposed a combined AFC with Probabilistic Neural Networks (PNNs) [65]. The proposed solution can lead to the following abilities: (a) incrementally learn from new training data sets; (b) freely add and remove activities in the system; and (c) remove the old data for new and updated training data. The system was implemented using a six-node BSN, and results were compared with three existing incremental learning methods, namely, Fuzzy ARTMAP (FAM), Radial Basis Function (RBF) networks, and probabilistic FAM. The experimental results show that average accuracies and execution times for incremental learning using five-fold technique are 91.3% and 264.3 s, respectively, while those obtained using leave-one-out technique are 89.2% and 325.4 s, respectively.
  The exploitation of clustering algorithms in PSDs highlights the issue of lightweight algorithms that have been explored in CAROMM [57]. The experiments were performed on Android-based mobile phones and tablets using Lightweight Clustering (LWC) algorithms proposed in OMM [12]. The results were reported in two parts: (a) data accuracy and (b) bandwidth and energy usage. The authors reported 300% energy gain and 17 times bandwidth gain with the same level of data accuracy as compared with raw data. Hence, it is setting a sound base for future mobile-based data mining systems.
  K-means clustering has been exploited in some studies. For example StressSense uses k-means to initialize means and variances of the GMM components [64]. Here, GMM is used with diagonal covariance matrix for classification of stress and neutral speech in proposed system. The classification decision is made using likelihood function p(X|λ) where X is a feature vector and λ(w; Σ; μ) is a GMM model with weight, mean, and covariance matrix parameters. The proposed system evaluated Akaike Information Criterion (AIC) for several predictive models on each subject. Resultantly the authors decided that 16 GMM components should be used for optimum classification results. However variance limiting techniques has been used to avoid over-fitting the training data. This variance limiting technique has been applied with standard expectation maximization (EM) algorithm to train the GMM speaker models [89].
  Another study used k-means clustering for a low-energy single-accelerometer-based complex activity recognition system [90]. As complex activity detection is more challenging because of the aperiodic and unpredictable nature of sensor data, the authors selected k-means clustering, where k = 10 is the optimal size. Multiple SVM-based fusion models for learning features were evaluated because of their superior support for classification and training. The reported results show that the proposed system achieves an average accuracy of 86.17%. Researchers proposed a sound classification system for mobile applications [63]. Here, the k-means clustering algorithm is applied for dimension reduction and hidden markov model (HMM) for classification. The average accuracy of sound classification in MobileSense is 95.31%.
  In other studies, TBC-based clustering algorithms were used for trajectory data mining and location prediction because of their support for historical data. TBC is used for efficient automatic navigation and location prediction in concentrative driving and regularly visited location history management [66,67]. According to a thorough literature reviewing, clustering-based algorithms, despite their major computational requirements, may be adapted more in future studies.
c) Frequent Pattern Mining
  Frequent Pattern Mining (FPM) is basically applied over I (set of items):{i_1_,…,i_n_}and T (set of transactions):{t_1_,..,t_n_}, where T ⊆ I. Transaction ID (TID) is used to uniquely identify a transaction in database [91]. T contains A (a set of items) iff A ⊆ T. The association rule A→B over two item sets A and B exists iff A ⊂ I and B ⊂ I and iff A∩B = ∅. The rule A→B contains the Transaction TID with minimum support s% for A→B and confidence c% for A∪B. Moreover, for a given set of Transactions D, the rule for minimum confidence (minconf) and minimum support (minsup) are specified by users, and all rules that support minconf and minsup are generated for D.
  These algorithms are generally designed to mine only frequent patterns and/or to find associations among different item sets. Overall research in frequent pattern mining varies from basic patterns to multilevel and multidimensional patterns, to extended patterns for data sets and streams. The extensive literature review shows that research in frequent pattern mining for PSD-based data mining systems is still at its initial stage. To the best of our knowledge, there are only two studies that purely use FPM algorithms in mobile commerce and activity recognition [27,92].
  In the first study, researchers proposed the Personal Mobile Commerce Pattern (PMCP-Mine) as part of the Mobile Commerce Explorer (MCE) framework to determine the personal shopping patterns of mobile users in m-commerce environments [92]. PMCP-Mine first mines the frequent mobile transactions from a user's local purchase data and then updates the local transaction database by removing the infrequent transactions. Finally, PMCP-Mine predicts new transaction patterns based on updated transaction patterns. The performance analysis of PMCP-Mine shows that execution time is incremental with the decrease in supported threshold value.
  In the second study, data mining technique based on emerging patterns (EP) was proposed in a complex activity recognition system that works at two layers [27]. In the first layer, the data are processed at BSN nodes and then transmitted to a mobile device for further processing. At the node level, lightweight algorithms are used for gesture recognitions and pattern-based real-time recognition algorithms are used in central portable devices. EP represents a set of frequent items in one class but a set of infrequent items in other classes. The assumption behind the EP-based technique is that instances containing EP items most likely belong to the corresponding EP class. The complexity analysis of the proposed algorithm shows that the time complexity of matching EP items with items stored in the class is O((*m*.*l* + *k*).*n*). Here *n* shows the length of input vector, *k* represents the total activities, *m* denotes the number of EPs, and *l* gives the average number of items in an EP. In addition, the space complexity of proposed algorithm is Θ(*m*.*l*) to hold the EPs. Performance analysis shows that the average recognition accuracy is 82.87%, the average recognition delay is 5.7 sensing periods, and the average utility is 0.81. Despite the high misdetection and false detection rate, the proposed algorithm performs better than the single-layer and HMM-based algorithms.
  Alternately, the exploitation of learning methods coupled with FPM algorithms is also gaining popularity in adaptive systems. For example, systems are using online incremental learning methods at first stage to continuously adapt with concept drift. In second stage, contextual correlations are discovered using proposed adaptive apriori algorithms [93]. It is worth to be noted that FPM algorithms and their combination with learning methods in resourceful environments are rigorously investigated but their utility in RCEs is still an unexplored research area. Therefore, the absence of literature on FPM techniques highlights the need for a detailed study to articulate the feasibility and performance of algorithms to find frequent patterns in RCEs.

### 3.3. Evaluation Criteria and Empirical Evidences

Data mining algorithms are usually evaluated with preset criteria for evaluation. The criteria used to evaluate the classifiers are usually accuracy, computational complexity, robustness, scalability, integration, comprehensibility, stability, and interestingness [62]. Accuracy is the basic criterion for the selection of classification algorithms, and classification time is the primary contributor in time complexity. Therefore, both of these metrics are usually considered in the performance evaluation of any classification algorithm.

Similarly, cluster evaluation is performed at two levels [64]. The Davis–Bouldin Index, Dunn Index, and Silhouette Coefficient are used for internal evaluation. Alternatively, Rand Measure, F-measure, Jaccard Index, Fowlkes–Mellow Index, confusion matrix, and mutual information are the external evaluation criteria for cluster analysis. The basic evaluation criteria for frequent pattern mining algorithms include time and computational complexities, accuracy, candidate counts, tree size, and number of frequent item sets [92]. Contrary to traditional data mining algorithms, PSD-based algorithms are evaluated with basic criteria because of their resource constraints. A detailed overview on data mining algorithms using these performance criteria for evaluation is presented in Table 6.

Space complexity (SP) evaluation is conducted to assess the computational (in terms of storage and memory) requirements of data mining algorithms. SP depends on the internal data structure and intermediate candidate generation of the algorithms. Similarly time complexity (TC) is evaluated to measure the execution time of data mining algorithms. Both SP and TC are measured using Big O notations. Note that Big O notations are used in the context of asymptotically bounded notations (also known as Big θ) because of resource constraints in PSDs.

Data mining algorithms deal with a massive amount of incoming data streams. Therefore, the number of instances that are accurately classified, clustered, or frequently counted is the primary contributor in the overall knowledge discovery process. The resultant patterns are classified as follows:

*True Positives* (*TP*): The number of instances correctly predicted as required.
*True Negatives* (*TN*): The number of instances correctly predicted as not required.
*False Positives* (*FP*): The number of instances incorrectly predicted as required.
*False Negatives* (*FN*): The number of instances incorrectly predicted as not required.

Various evaluation metrics are computed on the basis of these pattern classifications. A primary metric is accuracy (for classification) or Rand measure (for clustering algorithms), which is measured using Equation (1). The accuracy or Rand measure gives the percentage of correctly classified instances:

(1) $$\text{Accuracy}=\text{R}.\text{M}=\frac{TP+TN}{TP+FP+TN+FN}$$

As presented in Equation (2), precision determines the ratio of correctly detected/classified/clustered instances among all positive instances classified as positive instances:

(2) $$\text{Precision}=\frac{TP}{TP+FP}$$

Using Equation (3), sensitivity, which is also called recall value, is calculated to find the ratio of positive instances among all correctly detected instances:

(3) $$\text{Sensitivity}=\text{Recall}=\frac{TP}{TP+FN}$$

Alternatively, specificity metric, which is calculated using Equation (4), is used to determine the ratio of negative instances among all negative instances:

(4) $$\text{Specificity}=\frac{TN}{TN+FP}$$

F-score is another measure that combines the precision and recall values using slightly different equations, as presented in Equation (5) for classification and Equation (6) for cluster evaluation. Note that the β value is used to increase the impact of recall on the F-score. The greater the β values are, the lesser the precision impacts on the overall F-score of the underlying clusters:

(5) $$\text{F}-\text{Score}\;(\text{for classifiers})=2\times \frac{\text{precision}.\text{recall}}{\text{precision}+\text{recall}}$$

(6) $$\text{F}-\text{Score}\;(\text{for clusters})=\frac{\left(\beta^{2}+1)(\text{precision}.\text{recall}\right)}{\beta^{2}.\text{precision}+\text{recall}}$$

Miss detection, which is also called the false negative rate (FNR), is used to evaluate the percentage of data analyzed that produced false results. FNR is calculated using Equation (7):

(7) $$\text{Miss detection}\;(\text{False Negative Rate}\;(\text{FNR}))=\frac{FN}{TP+FN}$$

False detection, which is also called the false detection rate, is calculated using Equation (8) and it measures the percentage of data not analyzed at all:

(8) $$\text{False detection}\;(\text{False Detection Rate}\;(\text{FDR}))=\frac{FP}{TP+FP}$$

Aside from the basic performance metrics, a detailed evaluation of data mining algorithms could be done using a confusion matrix. The alternative terms used for confusion matrix are error matrix, contingency table for supervised learning, and matching matrix for unsupervised learning algorithms. Here, the obtained data about desired predictions and actual predictions are presented in matrix form. The diagonally highlighted cells, as presented in Table 7, accurately show the discovered patterns.

The Brier score (BS) [94] is another measure used to calibrate the prediction by finding the difference between probabilistic predictions and actual predictions. BS is calculated using Equation (9), where N is the total number of forecast predictions:

(9) $$BS=\frac{1}{N}\sum_{t=1}^{N}{\left(f_{t}-O_{t}\right)}^{2}$$

Clustering algorithms are evaluated with some extra metric. The patterns are evaluated internally (with data originally used for clustering) and externally (with unused data).

The Davies–Bouldin (DB) index is used for the internal evaluation of “n” number of clusters using Equation (10). The “C” values are the cluster centroids and the “σ” values are the average distance of all values from their respective centroids. The expression d(C_i_, C_j_) calculates the distance between two cluster centroids. Clustering algorithms with high inter-cluster and low intra-cluster distances have low DB index values. The algorithm with the lowest DB index is considered to be the best one:

(10) $$DB=\frac{1}{n}\sum_{i=1}^{n}\underset{i\neq j}{\max}\left(\frac{\sigma_{i}+\sigma_{j}}{d(C_{i},C_{j})}\right)$$

Similarly, the Dunn (D) index is used to find the density and separation of clusters. The D index, which is calculated using Equation (11), represents the ratio maximum intra-cluster distance d′(k) and minimum inter-cluster distance d(i,j):

(11) $$D=\underset{1\leq i\leq n}{\min}\left\{\underset{1\leq j\leq n,i\neq j}{\min}\left\{\frac{d(i,j)}{\underset{1\leq k\leq n}{\max}d^{\prime }(k)}\right\}\right\}$$

The silhouette coefficient is another metric used to compare the average distance among elements from the same cluster with the average distance among elements from other clusters. The measurement can be performed using Equation (12), which can be rewritten as Equation (13). Here, SC(n) is the silhouette coefficient, *x*(n) is the average distance measure within the same cluster, and *y*(n) is the lowest average distance measure of another cluster:

(12) $$\text{SC}(n)=\frac{y(n)-x(n)}{\max\{x(n),y(n)\}}$$

(13) $$\text{SC}(n)=[\begin{array}{c} 1-\frac{x(n)}{y(n)},if\;x(n)<y(n)\\ 0,if\;x(n)=y(n)\\ \frac{y(n)}{x(n)}-1,if\;x(n)>y(n) \end{array}$$

The Jaccard (J) index is a commonly used evaluation metric for external evaluation that measures the similarity between two datasets *X* and *Y*. The J-index is calculated using Equation (14):

(14) $$\text{J}(X,Y)=\frac{\left|X\cap Y\right|}{\left|X\cup Y\right|}=\frac{TP}{TP+FP+FN}$$

Similarly, the Fowlkes Mallow (FM) index measures the similarity between clustered and benchmarked results using Equation (15):

(15) $$\text{FM}=\sqrt{\frac{TP}{TP+FP}\times \frac{TP}{TP+FN}}$$

The Mutual Information measure is used to find the nonlinear similarity between different clusters using Equation (16), where *A* and *B* are different clusters, P(*a*, *b*) is the joint probability distribution, and *P*(*a*), *P*(*b*) is the marginal probability distribution of each cluster [95]:

(16) $$\text{I}(A;B)=\sum_{a\in A}\sum_{b\in B}P(a,b)\log\left[\frac{P(a,b)}{P(a)P(b)}\right]$$

The additional performance evaluation criteria of FPM algorithms include recoverability (similar to recall in classification and clustering), spuriousness, redundancy, number of patterns, significance, and robustness [96]. “*B*” is the set of base items defined as {b_1_, b_2_, …, b_n_} and “*F*” is the set of found patterns as {f_1_, f_2_, …, f_n_}. The recoverability metric is used to quantify the level at which the FPM algorithm can efficiently recover the base patterns. The F × B matrix is constructed first where each element in the matrix is represented as fb_ij_ at the ith row and jth column. Recoverability is computed using Equation (17):

(17) $$\text{Recoverability}\;(B)=\frac{\sum_{j=1\ldots |B|}\underset{i=1\ldots |F|}{\max}fb_{ij}}{\sum_{j=1\ldots |B|}|B_{j}|}$$

The spuriousness of a pattern determines the quality of a pattern by estimating the number of patterns that are not associated with ‘B’. Spuriousness can be measured using Equation (18):

(18) $$\text{Spuriousness}\;(F)=\frac{\sum_{i=1\ldots |F|}\left(|F_{i}|-\underset{j=1\ldots |B|}{\max}fb_{ij}\right)}{\sum_{i=1\ldots |F|}|F_{i}|}$$

Precision can be derived from spuriousness using Equation (19) as follows:

(19) Precision=1−spuriousness

The balance between spurious and useful patterns is measured by the significance metric. Significance, which is similar to the F-score in classification algorithms, is measured using Equation (20):

(20) $$\text{Significance}\;(F)=2\times \frac{\text{recoverability}\times \text{precision}}{\text{recoverability}+\text{precision}}$$

Redundancy is measured using Equation (21) as follows:

(21) $$\text{Redundancy}\;(F)=\frac{\sum_{i,j=1\ldots |F|}f\times f_{ij}-\sum_{i=1\ldots |F|}f\times f_{ij}}{2}$$

In addition with computational and performance evaluation measures of data mining algorithms, bandwidth consumption and energy consumption are the two key metrics that should be considered in PSD-based data mining algorithms [97]. The bandwidth consumption from a single device to ‘*m*’ servers in a given time period is defined in Equation (22) as follows:

(22) $${\text{Cost}}^{dt}=\text{totaldata}(\text{bytes})\times m$$

The bandwidth gain (BandwidthGain^dt^) after performing the cost analysis of PerDM algorithms in PSDs (*Patterns. Cost^dt^*) over cost of sending raw data (*Raw. Cost^dt^*) can be computed using Equation (23):

(23) $${\text{BandwidthGain}}^{dt}=\frac{\text{Raw}.\text{cos}t^{dt}}{\text{Patterns}.\text{cos}t^{dt}}$$

The overall cost of energy consumption Cost^eu^can be computed using Equation (24), where energy consumption during sensing and/or data acquisition ( ${\text{Cost}}_{s}^{eu}$), data processing ( ${\text{Cost}}_{pr}^{eu}$), and data transmission ( ${\text{Cost}}_{dt}^{eu}$) determine Cost^eu^:

(24) $${\text{Cost}}^{eu}={\text{Cost}}_{s}^{eu}+{\text{Cost}}_{pr}^{eu}+{\text{Cost}}_{dt}^{eu}$$

Energy gain (EngerGain*^eu^*) is computed using Equation (25), which shows the percentage of energy that could be saved after PerDM in PSDs *Patterns. Cost^eu^* compared with energy usage in raw data transmission *Raw. Cost^eu^* to external data mining platforms:

(25) $${\text{EnergyGain}}^{eu}=\frac{\text{Raw}.\text{cos}t^{eu}}{\text{Patterns}.\text{cos}t^{eu}}$$

## 4. Balancing Privacy, Personalization, and Security Using PerDM in PSDs

Currently, privacy- and security-related concerns are arising because of the proliferation and openness of mobile devices in pervasive and ubiquitous environments. The need for personalized services is also increasing because of massive data deluge in big data environments. Therefore, a well-designed PSD-based data mining system can play a significant role in the provision of privacy-preserving secure personalized services. The importance of such a system increases when dealing with highly personal data such as activities, emotions, banking information, and patients' physiological and genomic data sequences.

The designers of data mining systems need to create a balance to effectively meet three objectives: (1) the system must be highly secured but not at the cost of privacy and personalization; (2) the system must preserve privacy without compromising security and personalization; and (3) the system must provide effectively personalized services without undermining security and privacy. However, practically, a trade-off always exists among the three objectives in meeting the user requirements and enhancing the utility of the system.

**Figure 6.**
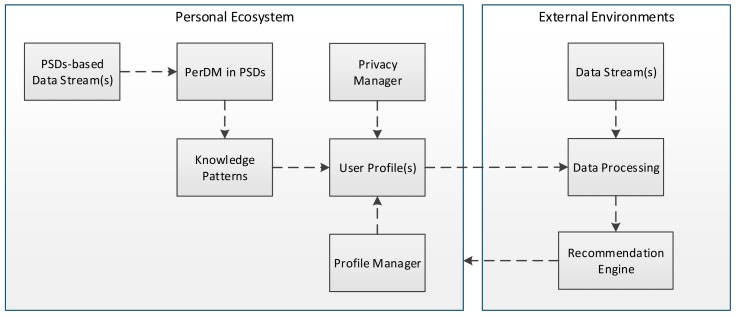
PSDs based user-centric personalization process.

A proper profiling of contextual information, preferences, and physiological and behavioral information at user premises can help to enhance privacy, security, and personalization. As the user is the core contributor and consumer of data in PEs, profile management at user premises will enhance his/her trust and privacy in the system. Some recent examples of user profiling in PSDs can be found in [92,98,99]. Data mining and machine learning techniques are good candidates for user profiling in ubiquitous and pervasive settings. For instance, personalized services for MCE presented in [92] are based on pattern mining, and the system presented in [100] provides personalization using decision tree. The proposed online multi-task learning algorithm in [101] deals with the problem of data sparseness in personalized activity recognition from a multi-person environment. Another recent application of frequent item-set mining for privacy-preserving and personalization is presented in [102]. Considering the success of PerDM in user-centric personalization in PSDs, we illustrate the process of user-centric personalization in external environments (Pervasive, Ubiquitous, or Big Data Ecosystem) in Figure 6 that uses PerDM in PSDs as a user profiling tool and in external environments as a personalization and recommendation engine.

The application of data mining and machine learning techniques is relatively new in PSD-based user-centric personalization. Some recent examples of personalized healthcare technologies can be found in activity recognition [103] and mobile-based systems [104]. However, to the best of our knowledge, no significant work that collectively covers the privacy, security, and personalization of big data streams in a ubiquitous environment has been conducted. Therefore, this research area should be explored. In conclusion, rapid development in PerDM algorithms and users' adoption of fine-grained PSDs are key evidence to establish the hypothesis on personalizing big data ecosystems in a private, secure, and cost-effective way.

### User-Centric Personalization Evaluation Model

**Figure 7.**
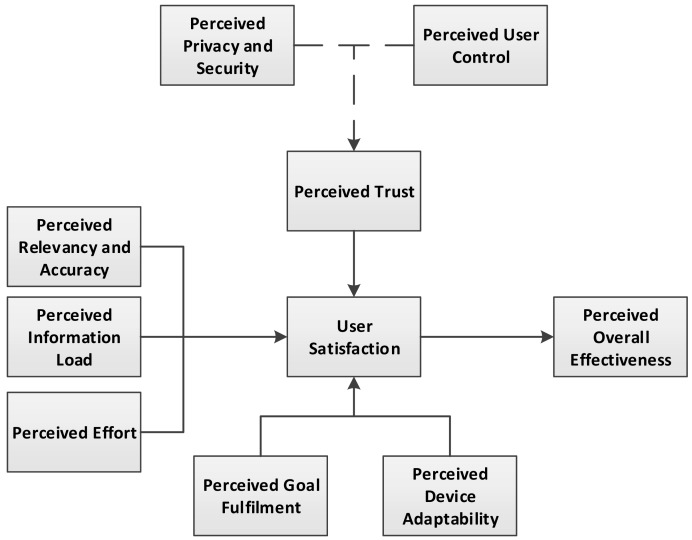
Evaluation model for PSDs based user-centric personalization [105].

According to a study [105], the overall effectiveness of user-centric personalization in PSDs is based on 10 constructs and. The connection between these constructs and the overall satisfaction and effectiveness is presented in Figure 7.

1. User satisfaction: The overall effectiveness of PSD is based on the strong connection between user satisfaction and user intention for using PSD. Therefore, an effective personalization strategy can enhance user satisfaction.
2. Perceived information load: The overwhelming amount of data causes information load in PSD, causing dissatisfaction and reluctance for PSD usage. Personalized services based on relevant information can reduce the information load and increase the overall usability of PSD.
3. Perceived relevance and accuracy: Personalization based on user profiles and contextual information can help present relevant and accurate information. Therefore, relevance and accuracy are directly related to the overall effectiveness of the system.
4. Perceived effort: The amount of effort required to use personalized contents is directly associated with user satisfaction because the effort labor required for exploring relevant results reduces the overall effectiveness of PSD.
5. Perceived security and privacy: The availability of sensitive personal information, such as financial and health data, needs a highly secure and privacy-preserving system. Therefore, privacy and security affect user satisfaction.
6. Perceived user control: The sensitivity of personal data and the need for personalization are the key drivers that make users lose control over their personal information. Therefore, personalized systems that provide sufficient user control over personal information enhance overall user satisfaction.
7. Perceived trust: Users produce and exchange different types of personal information. An effective mechanism for acquiring, processing, storing, and sharing of users' personal information at the service provider's end enhances users' trust in personalized systems.
8. Perceived goal fulfillment: Personalized systems are needed to fulfill user requirements for the functional and entertainment aspect. Goal fulfillment is directly linked to user satisfaction and intention for future use of personalized systems.
9. Perceive device adaptability: Personalized systems should be adaptable to user preference in multi-PSD environments. User interaction from each device should provide same level of service. Therefore, device adaptability directly affects user satisfaction.
10. Perceived overall effectiveness: The main construct of personalized systems is to measure the overall effectiveness for enhanced productivity, goal completion in a short time, and improved overall efficiency of users. Overall efficiency indicates the usability, personalization, and effectiveness of a system.

Here, we close the discussion on privacy-preserving, secure, and user-centric personalization using PSDs. We will look into the application areas and open research issues in the succeeding section.

## 5. Application Areas and Open Research Issues

According to existing literature, the provision of computational resources in fine-grained PSDs has opened new research horizons. The emergence of new application models and application areas has boosted relevant research. Therefore, PSDs are key enablers in some emerging research areas such as mobile commerce, humane activity recognition, mobile health, human–PSD interaction, physiological and behavioral monitoring and analysis, among others. The discussion on PerDM in PSDs is incomplete without highlighting some relevant application areas. A look into these application areas is presented in the following paragraphs.

The provision of customer-centric experience in terms of trust and usability is the key focal points in mobile commerce (m-commerce) environments. Diversity in m-commerce environments from in-store purchases to mobile and online purchases has stimulated customer-centric recommendation. The successful exploitation of data mining algorithms in m-commerce environments has opened new research horizons for PSD-based m-commerce environments [92]. Moreover, context management, privacy, personalization, and adaptation techniques are useful in attaining better customer experience [59,80]. In the future, PSD-based data mining systems will enable customers to manage and mine their data in PEs for privacy preservation and security. Moreover, the need for privacy-preserved anonymized data sharing is arising after the major shift of the banking industry from branchless to mobile banking, in which mobile payments and mobile account management have become the norm rather than the exception. Therefore, the local knowledge patterns discovered in PSDs can meet the privacy, personalization, and security requirements.

Humane Activity Recognition (HAR) from the recognition of simple ambulatory activities (e.g., walking, sitting, running, and sleeping) to complex activities (e.g., performing parallel or similar activities) is fueled by data mining techniques for sensory data sources [28,65,81,85]. Simple HAR systems are relatively easy to develop and deploy in PSDs, but the need for complex HAR systems still exists as an open research area. Moreover, the present HAR systems mostly perform periodic analysis or transmit data to remote DSPS for HAR. Therefore, future research should focus on real-time, online PSD-based HAR to reduce the need for external computational resources and scheduled HAR. Similarly, the scarcity of resources in PSDs limits classification-based HAR, thus creating research opportunities in two directions: (a) optimization of available classification algorithms and (b) exploitation of other data mining algorithms for activity recognition. For instance, existing algorithms should be optimized to meet the computational, energy, and bandwidth constraints. Clustering and FPM algorithms are also needed to be applied for feature diversity in HAR systems.

Mobile health (mHealth) is an emerging research area that focuses on the provision of healthcare services in remote and/or underdeveloped areas around the globe [106]. Initially, mobile phones were key enablers in mHealth technologies, but after the wide acceptance of IoT-enabled infrastructures, all types of PSDs are gaining popularity. For example, modern PSDs can sense patients' body temperature, heart rate, electrocardiogram, blood glucose level, and other physiological states [107,108]. The physiological data analysis techniques can help profile patients' daily health conditions [73]. Moreover, PSDs can perform behavioral analysis and categorize stress level and cognitive load [74,78]. The availability of physiological and behavioral monitoring can help in the reduction of service provision burden on the care providers' end and help in creating innovative patient-centric mHealth technologies.

Human–PSD interaction is another interesting area for the application of PerDM techniques. The single-user multi-device phenomenon in the modern era emphasizes the need for unobtrusive interaction and attention-oriented usage of PSDs in PEs. Moreover, the availability of a single-active screen in PSDs highlights the issue of intelligent adaptive user interfaces. PerDM algorithms in PSDs create a research opportunity to address these issues. For example, the analysis of application usage prediction can help develop personalized, interactive, and adaptive user interfaces [87].

In summary, we perceive that PerDM in RCEs will be able to leverage smartphones and wearable devices into a new era of ubiquitous sensing and pervasive computing. Moreover, the adoption of devices will open new research avenues to provide private, secure, and personalized ubiquitous services to users.

So far we discussed the design consideration, resource constraints, data mining schemes, and algorithms with evaluation criteria in relation to PerDM in RCEs using PSDs. In addition, we highlighted the issue of privacy, security, and personalization and discussed the role of PSDs based PerDM systems. Some application areas and future research opportunities were also discussed. In the following section, we conclude this study on the state of art of PerDM in RCEs.

## 6. Conclusions

The staggering growth in PSDs is a key enabler in PerDM in RCEs for personalization, privacy, and security at user premises. Moreover, the exploitation of data mining algorithms in PSDs enables the use of PEs for personal good. User-centric big data personalization is a concept with a wide range of application in health care, tourism, education, e-government, and smart cities, among others. It has immense potential in personalization for better patient, traveler, customer, student, and citizen experiences. For example, in case of personalized medicines, the idea of [109] is to provide the right treatment with the right dose and right drug at the right time. Therefore, the success of personalized medicine depends on the accurate diagnostics for targeted therapies.

PerDM in RCEs has a great potential to leverage these opportunities by monitoring user physiologies and diagnosing irregularities in patients' daily life data. The fusion of personal data in PSD-based systems with big health data creates an opportunity for patient-centric big data personalization. To the best of our knowledge, no practical user-centric PSD-based big data personalization system has yet been created. Therefore, we establish the hypothesis that the provision of personal data mining services in PEs can lead to highly secure, privacy-preserving personalized big data systems.

The vision can be met by enhancing the processing abilities of the current PerDM systems by focusing on execution models and data mining algorithms. The execution model can be optimized for cost and computation reduction by enabling maximum data processing inside PSDs and extending it to cloud-enabled data mining systems. Similarly, data mining algorithms should be designed to be scalable from small-scale, lightweight computations inside PSDs to compute-intensive tasks in cloud environments. Another important aspect is the enablement of privacy-preserving and personalized data processing in integrated processing modes to fully leverage heterogeneous computing devices uniformly.

## Figures and Tables

**Table 1.** Terminology.

**Term**	**Description**
PSDs	Personal Sensing Devices
RCEs	Resource-constrained Environments
PerDM	Personal Data Mining
PE	Personal Ecosystem
KDP	Knowledge Discovery Process
IoT	Internet of Things
EEU	Efficient Energy Utilization
BUC	Bandwidth Utilization Cost
BSN	Body Sensors Network
BLE	Bluetooth Low Energy
FPGA	Field Programmable Gate Array
DSPS	Data Stream Processing Systems
ID	Integration Device
OMM	Open Mobile Miner
SMA	Mobile Smart Achieve
FPM	Frequent Pattern Mining
SL	Supervised Learning
UL	Unsupervised learning
SSL	Semi-Supervised Learning

**Table 2.** Personal Data in PE.

**Data Source Type**	**Nature of Data Source**	**Data Source**	**Data Type**
Sensors	Physiological	Heart rate monitor	Numeric/Integer
		Blood Glucose Monitor	Numeric/Integer

	Physical activity	Accelerometer	Numeric/Floating point

	Environmental	Temperature	Numeric/Floating point
		Humidity	Numeric/Integer
		Air pressure monitor	Numeric/Floating point

	Navigational	GPS Location	Numeric/Floating point
		Compass	Text

User Interaction	Input data	On-screen keyboard	Text/Numeric
		Microphone	Audio
		Camera	Images/Video

Device-resident	Application Logs	Web browser logs	Text
		Application specific logs	Text

	Communication logs	Bluetooth scans	Text
		Wi-Fi Scans	Text

	User data	Contact List	Text
		Call Logs	Text
		SMS data	Text

**Table 3.** Large-scale DSPS.

**Platform**	**Purpose**	**Deployment**	**Prog. Language**	**Queries**
DataSift [37]	Twitter Stream Analysis	Cluster	Multiple	Filtering, Regular Expressions
Dremal [38]	Interactive *ad-hoc* querying	Cluster	Dremal query language	*ad-hoc* queries
ESper [39]	Complex Event Processing	In-Memory	Java, .Net	Online queries
IBM InfoSphere [40]	Stream processing	Cluster	Java	Online/*ad-hoc*
Kenesis [41]	Stream processing	Cluster	Java	Online queries
Hadoop Online Prototype [42]	Stream processing	Cluster	Java	Not Found
MOA [43]	Machine Learning	Various platforms	Java	Not Found
Microsoft Stream Insight [44]	Complex Event Processing	Server	.Net	Online queries
S4 [45]	Event processing	Cluster	Java	Online data processing
SAMOA [46]	Distributed machine learning	Can be integrated with S4 and Storm	Java	Not Found
Scikits.learn [47]	Machine learning	Programming Abstraction	Python, C++	Not Found
StreamDrill [48]	Stream processing	Not found	Not found	Top-K item counting
Storm [49]	Stream processing	Cluster	Multiple	Online

**Table 4.** Classification algorithms in PSDs.

**Study**	**Algorithm**	**Pre-Processing Technique**	**Learning**	**Max. Performance**	**Platform**
[72]	MNN	Feature extraction	Offline	Accuracy = 61.6%	Smartphone
[83]	NB with KDE	Captures RSSI values	Adaptive	Accuracy = 84%	Smartphone
[85]	J48	STFT and CWT	Offline	Accuracy = 97.2%	Smartphone
[78]	LDA	Feature extraction	Offline	Accuracy = 82.8%	MATLAB
[82]	HT, NB	80 (training) : 20 (test)	Online	Accuracy >50%	WEKA
[22]	C4.5	Feature extraction	Offline	Accuracy = 81.9%	Smartphone
[21]	ANN	Lag and autocorrelation plots, FFT and DCT	Offline	Accuracy with KDA [86] = 86.98%	MATLAB, Smartphone
[75]	SVM	Dimensionality and noise reduction, and feature extraction	Offline Online	Accuracy with KDA features = 99%	MATLAB, Smartphone
[76]	SVM	Feature extraction and Noise reduction	Offline	Accuracy = 98.85%	Smartphone
[84]	BN	Context inference module is used for adoption of BN	Online	Accuracy = 63%	Smartphone
[26]	J48	Feature extraction	Offline	Accuracy = 97.02%	Smartphone WEKA
[79]	QDA	Feature extraction	Offline	Accuracy = 95.8%	Smartphone
[73]	RF	Feature extraction	Offline	Accuracy = 80.3%	WEKA
[81]	NN	Feature extraction	Offline	Accuracy = 100%	WEKA
[77]	SVM	Feature extraction	Offline	SVM has best accuracy in almost all cases	PC, Smartphone
[59]	NB	Feature extraction	Online Adaptive	Accuracy = 86% ± 3.9%	ZTE Blade WEKA
[87]	kNN	Feature extraction	Offline	Recall = 95%	Smartphone
[74]	NN	Feature extraction	Offline	Accuracy = 100%	WEKA
[71]	MLP	Feature extraction	Offline	Accuracy = 50%	WEKA
[88]	J48 , LibSVM, AdaBoost, BN	Feature extraction	Offline	Average accuracy = 77.14%	Smartphone

**Table 5.** Clustering algorithms in PSDs.

**Study**	**Algorithm**	**Learning**	**Max. Performance**	**Platform**
[57]	Light-weight clustering	Online/offline	Energy gain = 300%, bandwidth gain = 17 times	Smartphone
[64]	*k*-means with GMM	Offline	Accuracy = 82.9%	Smartphone
[63]	*k*-means for dimension reduction	Offline	Accuracy = 95.31%	Smartphone
[65]	Adjustable fuzzy clustering with Probabilistic NNs	Offline/incremental	Accuracy = 91.3%	BSN
[66]	Time-based clustering	Online	Data reduction = 11*x*	Smartphone
[90]	*k*-means	Offline	Accuracy = 97.1%	Smartphone

**Table 6.** Evaluation criteria for data mining algorithms in PSDs.

**Study**	**Time Comp**	**Space Comp**	**Accuracy**	**Ener. Cons.**	**Precision**	**Recall**	**F-Score**	**Conf. Matrix**
[58]	-	-	√	-	-	√	√	√
[69]	-	-	√	-	-	-	-	√
[72]	√	-	√	-	-	-	-	√
[64]	-	-	√	-	-	-	-	-
[68]	-	-	√	-	-	-	-	-
[16]	√	-	√	√	-	-	-	√
[15]	-	-	√	√	-	-	-	√
[61]	-	-	√	-	-	-	-	-
[62]	√	√	√	-	-	-	-	-
[60]	-	-	√	-	-	-	√	-
[19]	√	√	√	√	-	-	-	√
[65]	-	-	√	-	-	-	-	-
[59]	-	-	√	√	-	√	√	-
[57]	-	-	√	-	-	-	-	-
[63]	√	-	√	√	-	-	-	-
[67]	-	-	√	-	√	√	√	-
[48]	√	√	√	√	-	-	-	-
[66]	√	-	√	-	√	√	√	-
[60]	-	-	√	-	√	-	-	-
[56]	-	-	√	-	√	√	√	√
[73]	-	-	√	-	-	-	-	√
[51]	√	-	√	-	-	-	-	-
[46]	-	-	√	√	-	-	-	-
[50]	-	-	√	-	-	-	-	-
[52]	-	√	-	-	-	-	-	-
[53]	√	√	TBC based algorithm was evaluated by No. of stay points and regions					
[49]	-	-	√	-	√	√	√	-
[74]	√	√	√	-	-	-	-	√

Note: The performance criteria used in respective studies is denoted by √, otherwise marked as -.

**Table 7.** Confusion matrix.

**Actual Predictions**					
**Desired Predictions**		Class1	Class2	Class3	Class4
	Class1				
	Class2				
	Class3				
	Class4				
